# Supplementary material for: Polybrominated Diphenyl Ether Serum Concentrations and Depressive Symptomatology in Pregnant African American Women
Source: Int J Environ Res Public Health. 2021 Mar 31;18(7):3614. doi: 10.3390/ijerph18073614 (PMC8037135; doi:10.3390/ijerph18073614)
Supplement: Supplementary file 1 [file ijerph-18-03614-s001.pdf]

Table S1. Demographic characteristics of an African American cohort 2014-2015 and comparison of subgroups.

Characteristic n (%)	Total cohort (n = 487)	Group A (n = 294)	Group B (n = 193)	P value
**Age (*M* ± *SD*)**	24.9 ± 4.7	25.3 ± 4.9	24.2 ± 4.4	*0.01
**Relationship status**				0.1
Not in relationship	104 (21.4)	69 (23.4)	35 (18.1)	
Relationship, not cohabitating	148 (30.4)	94 (32.0)	54 (28.0)	
Relationship, cohabitating	233 (47.8)	130 (44.2)	103 (53.3)	
**Education**				0.3
8th grade or less	1 (0.2)	1 (0.3)	0 (0)	
Some H.S	77 (15.8)	42 (14.3)	35 (18.1)	
Graduated H.S./GED	190 (39.0)	125 (42.5)	65 (33.7)	
Some college or tech	140 (28.7)	77 (26.2)	63 (32.6)	
Graduated college	50 (10.3)	30 (10.2)	20 (10.4)	
Graduate school or degree	29 (6.0)	19 (6.5)	10 (5.2)	
**Parity**				0.2
0	240 (49.3)	140 (47.6)	100 (51.8)	
1	140 (28.7)	82 (27.9)	58 (30.0)	
2+	107 (22.0)	72 (24.5)	35 (18.1)	
**Insurance**				0.06
Medicaid	382 (78.4)	234 (79.6)	148 (76.7)	
LIM<100%	180 (46.9)	84 (35.9)	96 (64.9)	
RSM≤ 200%	202 (52.9)	150 (64.1)	52 (35.1)	
Private	105 (21.6)	60 (20.4)	45 (23.3)	
**Body mass index**				0.8
Underweight (<18.5)	18 (3.7)	10 (3.4)	8 (4.2)	
Normal (18.5 – < 25)	192 (39.4)	112 (38.1)	80 (41.5)	
Overweight (25 – < 30)	102 (20.9)	63 (21.4)	39 (20.2)	
Obese (≥ 30)	175 (35.9)	109 (37.1)	66 (34.2)	
**Edinburgh Depression Scale Total Score (*M* ± *SD*)**	7.2 ± 5.2	7.5 ± 5.6	6.8 ± 5.2	0.2
**Drinks alcohol in first trimester**	27 (5.7)	19 (6.9)	8 (4.2)	0.2
**Smoked tobacco ever**	67 (14.1)	46 (16.4)	21 (10.9)	0.09
**Smoked MJ in first trimester**	104 (22.1)	71 (25.5)	33 (17.3)	*0.04

*Age and smoked marijuana in first trimester means of group A and group B are statistically significant ($p \leq 0.05$). Equal variances are assumed.

Table S2. Limits of detection (LOD) obtained with GC-MS for PBDE concentrations (pg/mL).

Metabolite	LOD
PBDE 47	3.13
PBDE 85	78.13
PBDE 99	7.81
PBDE 100	3.13
PBDE153	78.13
PBDE 154	78.13

Table S3. Comparison of depressive symptoms among African American women at 8-14 weeks and 24-30 s weeks gestation.

Time point	N	EDS Score mean ± SD	High risk of depression n (%)	Difference in Mean P Value
**8–14 weeks**	193	6.82 ± 5.24	52 (27)	0.28
**24–30 weeks**	160	6.20 ± 5.52	39 (20)	

EDS = Edinburgh Depression Scale

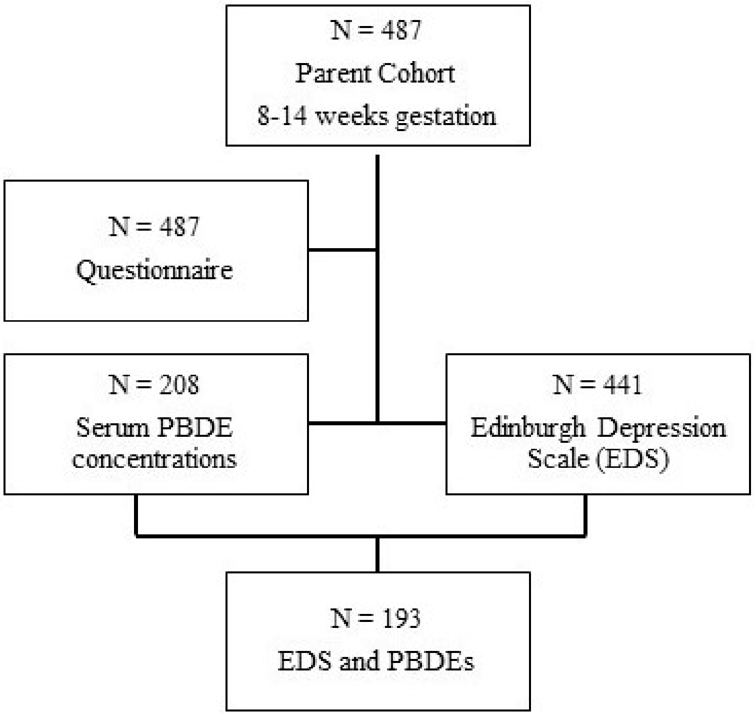

Figure S1. Flow diagram of study selection (N = 193) from parent cohort sample.
